# Pyoderma Gangrenosum With Severe Ankle Ankylosis Complicated by Rheumatoid Arthritis Successfully Treated Using Anti-tumor Necrosis Factor: A Case Report

**DOI:** 10.7759/cureus.75614

**Published:** 2024-12-12

**Authors:** Takaaki Nagase, Yuya Takakubo, Yoshihiro Wanezaki, Masashi Aso, Michiaki Takagi

**Affiliations:** 1 Department of Orthopaedic Surgery, Yamagata University Faculty of Medicine, Yamagata, JPN; 2 Orthopaedics and Rehabilitation, Yamagata University Hospital, Yamagata, JPN

**Keywords:** ankylosis, anti-tumor necrosis factor, pyoderma gangrenosum, rheumatoid arthritis, ulcer

## Abstract

Pyoderma gangrenosum (PG) is a rare dermatosis characterised by necrotic ulcers with a predilection mainly for the lower legs.

We report a case of a 67-year-old man with PG and severe ankle ankylosis complicated by rheumatoid arthritis (RA) treated with anti-tumor necrosis factor, adalimumab (ADA). He was referred to our hospital because his right ankle showed severe ulcers and blackening. X-rays of the right ankle and foot showed bone and joint destruction with ankylosis. Initially diagnosed with stasis dermatitis, he was treated, but his condition became increasingly worse. A skin biopsy revealed neutrophilic invasion of the skin tissue, and final diagnosis was PG complicated with RA and ankylosing joint. ADA therapy improved the patient’s skin condition and reduced the right ankle pain, although severe right ankle ankylosis progressed during the last follow-up.

PG should be considered as a differential in patients with RA and refractory skin ulcers.

## Introduction

Pyoderma gangrenosum (PG) is a dermatosis characterised by gangrenous ulcers with a predilection mainly for the lower legs [1]. PG is associated with ulcerative colitis (UC) and rheumatoid arthritis (RA) [2]. Despite being a well-recognised condition, clinicians often fail to make an early diagnosis of PG. We report a case of ankle arthritis with refractory skin ulceration in which we delayed aggressive PG treatment because infection was initially suspected, and later adalimumab (ADA) treatment improved the skin findings but failed to prevent progression of the ankle arthritis.

## Case presentation

A written informed consent for this case report was obtained from the patient, and this research was approved by the authors’ affiliated institutions.

**Figure 1:**
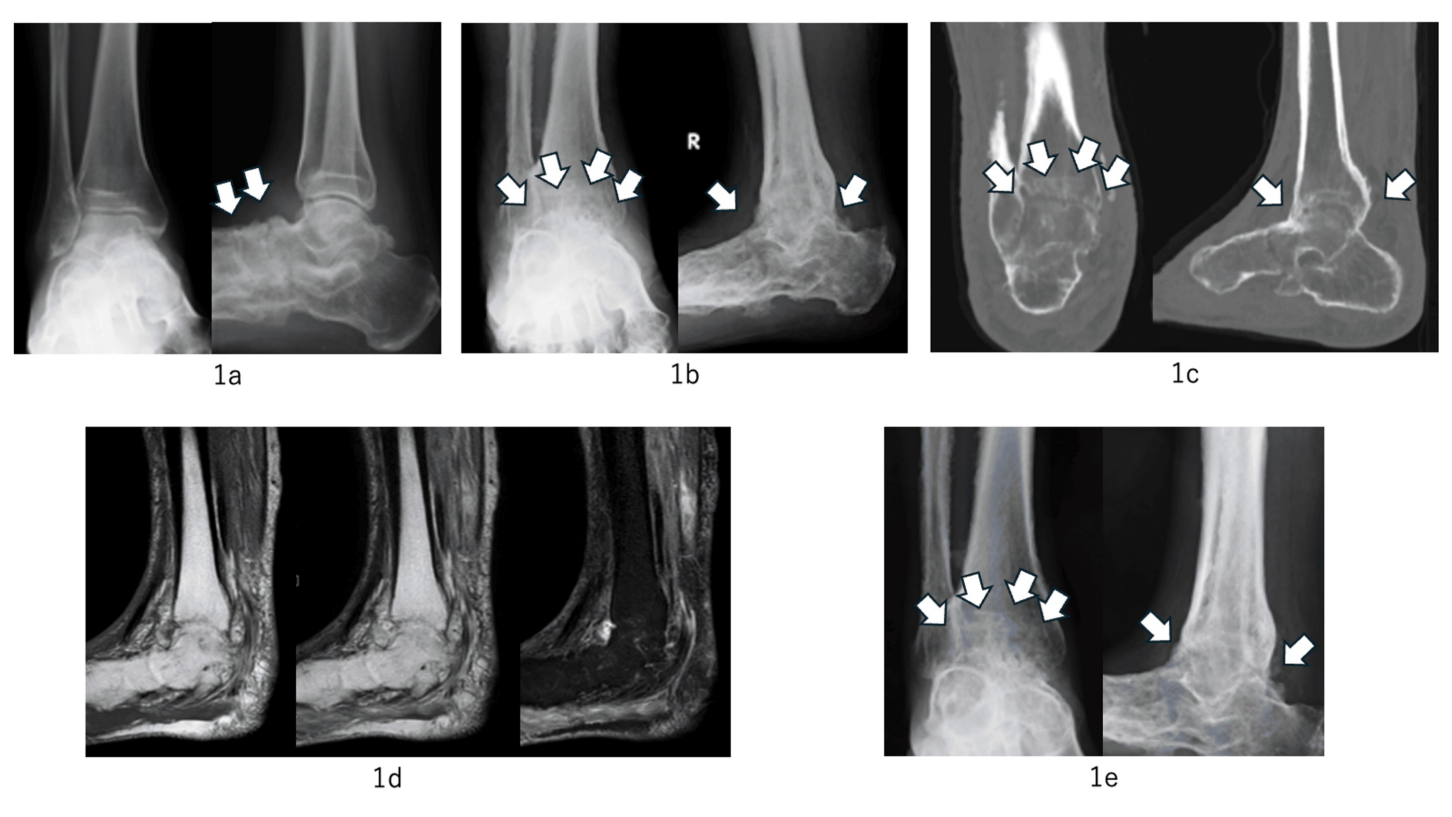
Image findings X-rays of the right ankle 15 years ago. There was no destruction of the ankle joint 15 years ago, but bone erosion and joint space narrowing of the midfoot were seen (1a, point out joint space narrowing of the midfoot, white arrow). X-rays and computed tomography (CT) of the right ankle when he referral to our hospital. bone and joint destruction were observed with severe ankle ankylosis (1b; X-ray, point out ankle ankylosis, white arrow. 1c; CT, left; sagittal view, right; coronal view, point out ankle ankylosis, white arrow.). Magnetic resonance imaging (MRI) of the right ankle upon referral to our hospital. MRI revealed no abscesses and no signal changes which were indicative of infection. (1d, left; T1WI sagittal, center; T2WI sagittal, right; DIWI sagittal) Upon the last examination, the joint destruction and ankylosis of the ankle slightly progressed compared to the initial evaluation at our hospital (1e, point out ankle ankylosis, white arrow.).

**Figure 2:**
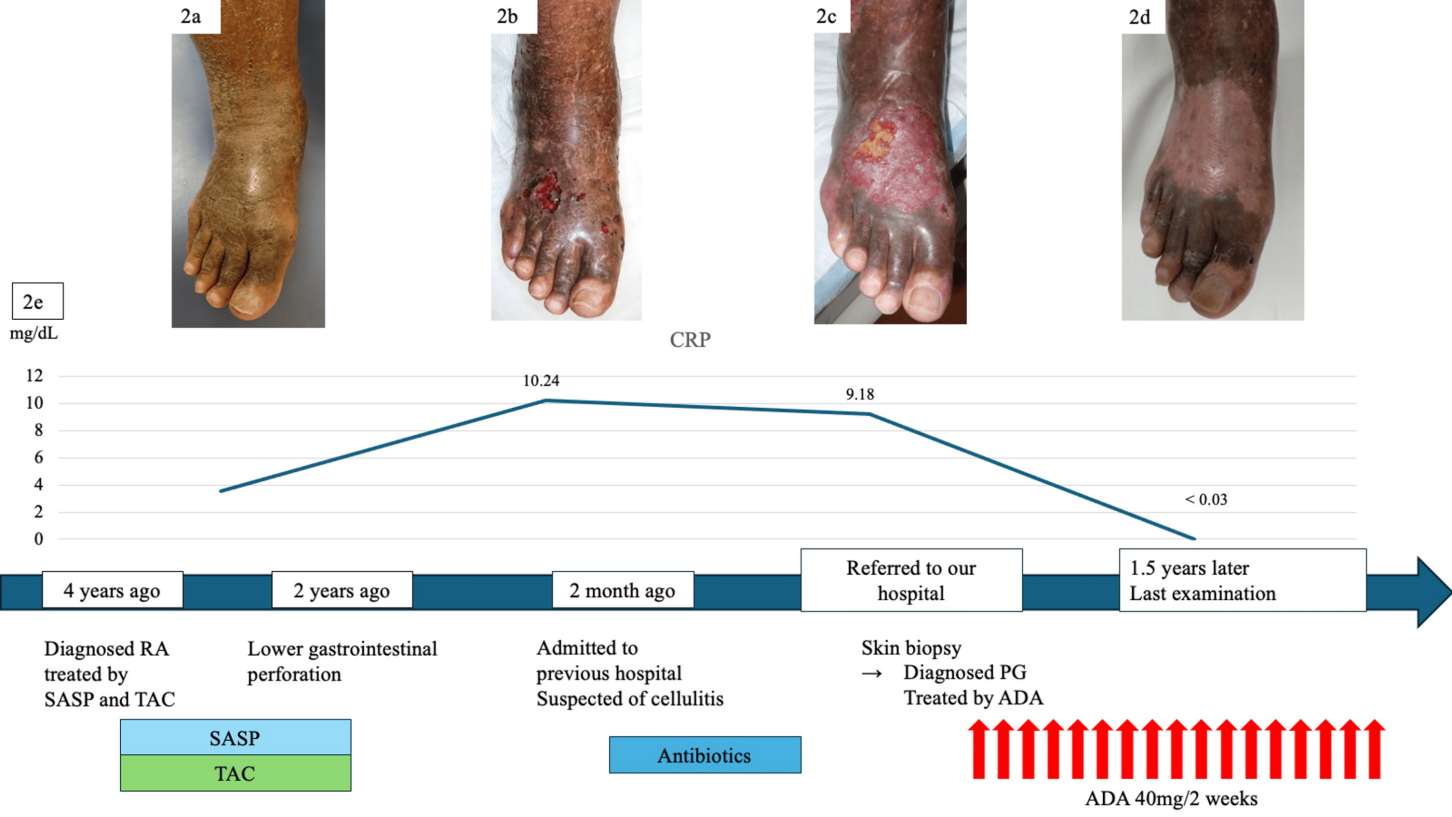
Macroscopic view of the ankle and foot Therapeutic course and macroscopic view of the ankle and foot four years ago. He was diagnosed with rheumatoid arthritis (RA) and treated by salazosulfapyridine (SASP) and tacrolimus (TAC). The right ankle and foot had blackening, but no ulcers (2a). Two years ago, because he had lower gastrointestinal perforation, he had to quit taking SASP and TAC. Two months before referring to our hospital, his right leg had developed a severe ulcer and blackening (2b). He was admitted to previous hospital and was treated by antibiotics because he was suspected of cellulitis. Laboratory data showed C-reactive protein (CRP) was 10.24 mg/dL. The right ankle and foot had severe ulcers and more blackening upon referral to our hospital (2c). We performed skin biopsy and treated for pyoderma gangrenosum (PG) by anti-tumor necrosis factor therapy, adalimumab (ADA). During the last examination, the skin condition of his ankle and foot improved significantly by ADA (2d). CRP was improved to < 0.03 mg/dL (2e).

**Figure 3:**
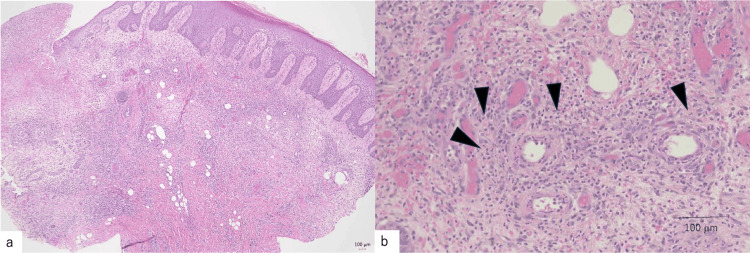
Pathological findings of the right ankle skin. Skin biopsy showed neutrophilic invasion (black arrow) of the skin tissue and ulcer. There were no findings of vasculitis or infection (a, b, light microscopic images were stained with hematoxylin-eosin).

The patient was a 67-year-old male. He was a farmer, and his comorbidity was hypertension. He experienced right ankle pain. Fifteen years ago, he was diagnosed with right ankle osteoarthritis at another hospital, wherein bone erosion and joint space narrowing were seen in the midfoot on X-ray (Figure 1A). Eight years ago, he sought to consult for left knee pain, and blood tests showed increased levels of C-reactive protein (CRP) (3.55 mg/mL) and matrix metalloproteinase-3 (MMP-3) (357 ng/ml). X-ray revealed advanced erosion of the talonavicular joint and cuneonavicular joint compared to that 15 years ago, and positron emission computed tomography showed accumulation of 18 F-fluorodeoxyglucose at the left knee and right ankle joints and revealed a diagnosis of oligoarthritis (left knee and right ankle). Four years ago, he underwent arthroscopic synovectomy for left knee pain in the previous hospital. Pathology showed only synovitis with lymphoid aggregation. Although rheumatoid factor (RF) was 29 ng/dL and anti-citrullinated protein antibody (ACPA) was negative (<0.5 U/mL), we diagnosed RA by pathological findings (inflammatory synovitis with lymphoid aggregation) and physical findings (left knee and right ankle arthritis). He was treated by salazosulfapyridine and tacrolimus (Figure 2A). After two years, however, treatment was stopped due to lower gastrointestinal perforation. The findings of inflammatory bowel disease (IBD) were not found by any imaging and pathological examinations of his abdominal area. Two months before referring to our hospital, his right leg had developed a severe ulcer and was blackened (Figure 2B). Suspecting cellulitis in his right leg, he was admitted to the previous hospital, despite antibacterial treatment, his lower leg condition was not improved. After that, he was referred to our hospital as refractory cellulitis. His right ankle had severe ulcers and more blackening (Figure 2C). His right ankle and foot showed worsening bone and joint destruction with severe ankle ankylosis (Figure 1B, 1C). Because his ankle had a limited range of motion and he could not walk well, he was wheelchair-bound during hospitalization. Laboratory data revealed white blood cell counts of 10,590/μL, CRP of 10.24 mg/mL and MMP3 of 265.6 ng/ml, RF was 74 ng/dL, whereas ACPA and antinuclear antibody were negative (Table 1). On magnetic resonance imaging, there was no abscess and no signal changes suggestive of infection in the right ankle (Figure 1D). Bone scintigraphy showed small hot spots in his right foot, but there were no findings of osteomyelitis. When he was referred to our hospital, we consulted a dermatologist in our hospital regarding the ulcer. They commented that stasis dermatitis was suspected. Treatment was initially initiated as stasis dermatitis. The wound was flushed with running water and sulphadiazine silver was applied daily. However, the patient experienced repeated exacerbations of disease activity. Skin biopsy performed by dermatologists showed neutrophilic invasion of the skin tissue and ulcer, with no findings of vasculitis or infection (Figure 3). PG was diagnosed as a complication of RA due to the progressive joint destruction and skin biopsy findings. We started subcutaneous injections of ADA at 40 mg per two weeks. After the initiation of ADA, his skin conditions and ankle pain significantly improved (Figure 2D). However, ankylosis of his ankle was worse gradually compared to the X-ray at his first visit to our hospital (Figure 1E).

**Table 1:** Results of blood examinations upon referral to our hospital

	Measured value		Normal ranges
Leukocytes	10,590	/µl	4000-9000
Neutrophils	9,400	/µl	1,800-7,500
Red blood cells	420	× 10^4^/µl	400-500
Haemoglobin	9.2	g/dL	13-17
Platelets	44.1	× 10^4^/µl	11-37
C-reactive protein	10.24	mg/dL	0-0.3
Matrix metalloproteinase-3	246.5	ng/dL	36.9-121
Blood urea nitrogen	8	mg/dL	7.5-20
Creatinine	0.67	mg/dL	0.6-1.1
Aspartate transaminase	16	U/L	8-40
Alanine transaminase	27	U/L	5-40
Alkaline phosphatase	246	U/L	38-113
Total bilirubin	0.3	/μL	0.3-1.2
γ-Glutamyl Transpeptidase	27	U/L	10-68
Rheumatoid factor	74	ng/dL	<15
Anti-cyclic citrullinated peptide antibody	0.8	U/mL	<4.5
The antinuclear antibody	<40		<40
Single-stranded DNA	0.9	IU/mL	<7
Double-stranded DNA	0.9	IU/mL	<10
Cytoplasmic antineutrophil cytoplasmic antibody	<0.6	U/mL	<3.5
Perinuclear antineutrophil cytoplasmic antibody	<0.2	U/mL	<2
CH50	70.5	U/mL	30-45

## Discussion

We report a case of PG complicated with RA and severe ankle ankylosis. ACPA was negative (0.8 U/mL), but RF was positive (74 ng/dL) and based on pathology (inflammatory synovitis with lymphocyte aggregation) and physical findings (arthritis in left knee and right ankle, not symmetrical), we diagnosed RA. Although ulceration associated with chronic arthritis cannot be denied, we diagnosed an ulceration of his foot as due to PG, considering the concomitant RA, the significant effectiveness of ADA therapy for ulceration, and pathological findings of the skin.

PG is a reactive, aseptic, and inflammatory dermatosis that falls under the spectrum of neutrophilic dermatoses [3,4]. PG is classified into five subtypes (ulcerative, bullous, pustular, vegetative, and peri-stomal), among which the ulcerative type is the most common, accounting for approximately 85% [3]. The incidence of PG is 0.3 and 0.63 per 10,000 patients in Japan [4,5], and in the UK [6], respectively. PG often occurs at 20-60 years of age and is slightly more common in women [7]. It frequently affects the lower legs, although PG can be present at any site [5,8]. PG is reported as one of the complications of RA, haematologic disease and in IBD, especially, IBD is the most frequent [3,4,9]. In fact, the risk of developing PG is tripled in patients with RA compared to the general population [2,8]. In this case, there were no signs of IBD, even though the patient had suffered from a lower gastrointestinal perforation at the previous hospital.

Despite being a well-recognized condition, clinicians often fail to make an early diagnosis of PG. This may be because there are no clinical or pathological criteria for PG. PG is a diagnosis of exclusion; it is therefore important to differentiate it from other diseases with similar symptoms and lesions [3,8]. Skin biopsy is important, although its purpose is to exclude other causes of cutaneous ulceration, rather than to make a definitive diagnosis. Nevertheless, it is especially useful to exclude malignancy, vasculitis and infections [10]. In this case, a biopsy revealed neutrophilic infiltration and ulceration of the epidermis and dermis. Although rheumatoid vasculitis causing ulceration is a possible differential diagnosis, there was no initial suspicion of vasculitis.

In the treatment of PG, it’s important to take care of the affected area [3,8,9]. Although these are often debrided by providers not familiar with the diagnosis, debridement makes PG worse and is strictly contraindicated. More severe cases with deeper and more widespread ulcers are often managed with antirheumatic drugs such as glucocorticoids, cyclosporin A and tumor necrosis factor (TNF) inhibitors, especially ADA and infliximab (IFX) [3,4]. Wound healing is reportedly faster in treatment with TNF inhibitors (e.g., IFX and ADA) than with oral corticosteroids alone [11]. An excellent response to TNF inhibitor treatment has been documented several times in patients with PG [1,9]. Tada et al. reported the case of a patient with PG complicated by RA who was treated using IFX therapy, and thus it may be another treatment option for these cases [12]. In addition, it was reported that TNF-α and interleukin 8 (IL-8) are produced in excess in the skin of patients with PG compared to normal skin [13]. Thus, biologics that target these cytokines may be effective in PG. A clinical trial and prospective study on the clinical efficacy of a biological agent with ADA in PG in Japanese patients achieved PG area reduction, and no adverse events specific to ADA were observed [14]. On the other hand, since the occurrence of PG has been reported as one of the paradoxical reactions after TNF inhibitor administration in a small number of cases, careful follow-up is necessary in the future [15]. Recently, Iliescu et al. reported a case where a patient stopped treatment for PG. This caused the disease to spread quickly and severely, leading to toe amputation and exposure of important tissues in the ulcers. They highlighted the need to follow treatment strictly and monitor the condition carefully [16].

In this case, the patient was initially treated with antibiotics for cellulitis but didn't improve in the previous hospital. After being referred to our hospital, we started the treatment for cellulitis and stasis dermatitis according to the dermatologist consultation, although there was no significant improvement. Patient had oligoarthritis (right foot and left knee) without the typical symptoms of RA including hand arthritis or symmetry joint disorders. The patient was diagnosed with PG complicated with RA based on the findings of images and ankle skin biopsy and started subcutaneous injection of ADA. Because the diagnosis of his ulcer was not confirmed completely at the start of ADA therapy, and the possibility of an infection could not be ruled out, the loading dose of ADA was not administered. After the initiation of ADA administration, skin symptoms and pain improved with the results of inflammatory response of blood test immediately. So, the dosage of ADA was not increased further.

ADA therapy improved the patient's skin condition and reduced her right ankle pain, but because this case was initially treated as an infection, ankle ankylosis had progressed by the time the patient was referred to our clinic. In addition, even though there has been improvement in terms of skin findings, melanization (hyperpigmentation) and thickening of the skin remained. At the last observation, he had no pain and complaints for his right ankle, but the ankle ankylosis had advanced more.

## Conclusions

We report a case of severe ankle ankylosis with PG complicated by RA. We delayed aggressive treatment for severe skin ulcers with ankle arthritis because of suspected infection. ADA therapy improved the patient's skin condition, and reduced the right ankle pain, but did not prevent progression of the ankle ankylosis. Therefore PG should be considered as a differential in patients with RA and refractory skin ulcers.
